# Bi-directional regulation of AIMP2 and its splice variant on PARP-1-dependent neuronal cell death; Therapeutic implication for Parkinson's disease

**DOI:** 10.1186/s40478-023-01697-5

**Published:** 2024-01-03

**Authors:** Min Hak Lee, Ki-Hwan Um, Seok Won Lee, Ye Ji Sun, Da-Hye Gu, Young Ok Jo, Sung Hyun Kim, Wongi Seol, Hyorin Hwang, Kyunghwa Baek, Jin Woo Choi

**Affiliations:** 1 Department of Pharmacology, College of Pharmacy, Kyung Hee University, Seoul, 02447 Republic of Korea; 2 Department of Biomedical and Pharmaceutical Science, Graduate School, Kyung Hee University, Seoul, 02447 Republic of Korea; 3 Department of Regulatory Science, College of Pharmacy, Kyung Hee University, Seoul, 02447 Republic of Korea; 4 Department of Neuroscience, Graduate School, Kyung Hee University, Seoul, 02447 Republic of Korea; 5 Generoath Ltd., Seoul, 04168 Republic of Korea; 6 Department of Physiology, School of Medicine, Kyung Hee University, Seoul, 02447 Republic of Korea; 7 InAm Neuroscience Research Center, Sanbon Medical Center, College of Medicine, Wonkwang University, Sanbonro 321, Gunposi, Gyeonggido 15865 Republic of Korea; 8 Department of Pharmacology, College of Dentistry and Research Institute of Oral Science, Gangneung-Wonju National University, Gangneung, Gangwon-Do 25457 Republic of Korea

**Keywords:** AIMP2 splicing variants, PARP-1, Parkinson’s disease, Adeno-associated virus, Gene therapy

## Abstract

**Background:**

Parthanatos represents a critical molecular aspect of Parkinson's disease, wherein AIMP2 aberrantly activates PARP-1 through direct physical interaction. Although AIMP2 ought to be a therapeutic target for the disease, regrettably, it is deemed undruggable due to its non-enzymatic nature and predominant localization within the tRNA synthetase multi-complex. Instead, AIMP2 possesses an antagonistic splice variant, designated DX2, which counteracts AIMP2-induced apoptosis in the p53 or inflammatory pathway. Consequently, we examined whether DX2 competes with AIMP2 for PARP-1 activation and is therapeutically effective in Parkinson’s disease.

**Methods:**

The binding affinity of AIMP2 and DX2 to PARP-1 was contrasted through immunoprecipitation. The efficacy of DX2 in neuronal cell death was assessed under 6-OHDA and H2O2 in vitro conditions. Additionally, endosomal and exosomal activity of synaptic vesicles was gauged in AIMP2 or DX2 overexpressed hippocampal primary neurons utilizing optical live imaging with VAMP-vGlut1 probes. To ascertain the role of DX2 in vivo, rotenone-induced behavioral alterations were compared between wild-type and DX2 transgenic animals. A DX2-encoding self-complementary adeno-associated virus (scAAV) was intracranially injected into 6-OHDA induced in vivo animal models, and their mobility was examined. Subsequently, the isolated brain tissues were analyzed.

**Results:**

DX2 translocates into the nucleus upon ROS stress more rapidly than AIMP2. The binding affinity of DX2 to PARP-1 appeared to be more robust compared to that of AIMP2, resulting in the inhibition of PARP-1 induced neuronal cell death. DX2 transgenic animals exhibited neuroprotective behavior in rotenone-induced neuronal damage conditions. Following a single intracranial injection of AAV-DX2, both behavior and mobility were consistently ameliorated in neurodegenerative animal models induced by 6-OHDA.

**Conclusion:**

AIMP2 and DX2 are proposed to engage in bidirectional regulation of parthanatos. They physically interact with PARP-1. Notably, DX2's cell survival properties manifest exclusively in the context of abnormal AIMP2 accumulation, devoid of any tumorigenic effects. This suggests that DX2 could represent a distinctive therapeutic target for addressing Parkinson's disease in patients.

**Supplementary Information:**

The online version contains supplementary material available at 10.1186/s40478-023-01697-5.

## Background

Poly(ADP-ribose) polymerase-1 (PARP-1) serves as a double-edged sword in cellular processes, playing pivotal roles in both cell survival and death. As a key facilitator of DNA damage repair via polyADP-ribosylation (PARylation), PARP-1 contributes to cellular survival [18]. Nevertheless, its overactivation results in cytosolic NAD+ depletion, poly-ADP-ribose (PAR) formation, mitochondrial apoptosis-inducing factor (AIF) release, nuclear AIF translocation, and ultimately, parthanatos-mediated cell death[26]. Thus, while PARP-1 inhibition may promote cell lethality through excessive DNA damage in cancer therapy, prudent PARP-1 suppression could offer a therapeutic avenue to regulate abnormal neuronal death in conditions such as Parkinson's disease (PD) and ischemia [56].

PD, a neurodegenerative disorder, is characterized by dopaminergic neuronal loss in the substantia nigra pars compacta (SNpc) [52]. With aging, PD prevalence increases, and affected individuals' brain exhibits abnormal protein aggregates called Lewy bodies, composed of parkin (an E3 ubiquitin ligase component) and α-synuclein. Although the etiology of PD remains enigmatic, parkin mutations have been linked to autosomal recessive juvenile parkinsonism (AR-JP) [32, 49]. In sporadic PD, the more common form, parkin often becomes inactivated via S-nitrosylation, oxidative and dopaminergic stress, and c-Abl phosphorylation [17].

Given parkin's role as an SCF-like ubiquitin ligase complex component, potential substrates degraded by parkin, such as aminoacyl-tRNA synthetase interacting multifunctional protein 2 (AIMP2, also known as p38 or JTV-1) [15, 52], have emerged as promising therapeutic targets for PD. Elevated AIMP2 expression in post-mortem PD patient’s brain and parkin knockout mice suggests its critical involvement in PD pathophysiology [33]. AIMP2 is known to respond to genetic damage through direct interaction with p53 and to promote TNF-α-dependent cell death via TNF receptor-associated factor 2 (TRAF2), possibly acting as a multidirectional apoptotic factor [12]. However, the exact mechanism underlying AIMP2's influence on PD remains elusive. Recent findings by Lee et al. revealed that AIMP2 overexpression directly activates PARP-1 and triggers dopaminergic neuronal cell death in AIMP2 transgenic mice [38]. Consequently, AIMP2-induced PD implicates PARP-1-mediated parthanatos as a central driver of disease progression.

Although AIMP2 serves as the upstream regulator of PARP-1 activation, its undruggability—owing to its non-enzymatic and housekeeping scaffold properties [45]—precludes it as a viable PD therapeutic target. Instead, PARP inhibitors hold promise as potential PD therapeutics by suppressing AIMP2-aberrantly activated PARP-1. However, the ability of PARP inhibitors to cross the blood–brain barrier remains uncertain [22]. Furthermore, considering PARP-1's crucial role in DNA damage sensing and repair, long-term treatment with PARP inhibitors may provoke significant adverse effects in PD patients [7].

To identify a safe and specific target for treating PD, we assessed AIMP2 as a potential candidate for mitigating dopaminergic cell death. In our previous research, we discovered that the AIMP2 splice variant, DX2, which lacks exon 2 of the full-length AIMP2, competes with AIMP2 for p53 binding [11]. Remarkably, we observed distinct functional characteristics between full-length AIMP2 and its splice variant DX2 in TNF-α signaling. In ovarian epithelial cells, such as SKOV3 and A2780, AIMP2 elicited apoptosis by degrading TRAF2 and inhibiting NF-κB. In contrast, DX2 facilitated cell survival by stabilizing TRAF2 in response to TNF-α treatment [13]. These findings propose that DX2 might function as an endogenous antagonist of proapoptotic full-length AIMP2, maintaining a delicate equilibrium between cellular survival and death. If DX2 can effectively impede AIMP2’s function in PD, it may mitigate dopaminergic neuronal cell death. Our study demonstrates that DX2 proficiently suppresses AIMP2 function by modulating PARP-1 activation.

Central nervous system (CNS) disorders display convoluted pathophysiology and elaborate molecular mechanisms, making drug delivery to target cells arduous due to the CNS's unique anatomical features [4]. As a result, the pursuit of more efficacious treatment strategies to supersede existing drugs persists. The current study endeavors to evaluate the potency of a recombinant adeno-associated virus (AAV) vector system in delivering DX2, consequently inhibiting AIMP2 function in PARP-1 overactivation both in vitro and in vivo.

## Materials and method

### Cell lines and reagents

SK-SH5Y, SK-N-SH, and N2A cells were obtained from the Korean Cell Line Bank (KCLB, Seoul, Korea). Cells were grown in RPMI-1640, and supplemented with 10% fetal bovine serum (FBS) and 1% penicillin–streptomycin (HyClone, PA, USA). The transient transfection of myc-tagged or GFP-tagged parkin, AIMP2, DX2, HA-tagged ubiquitin, and empty vector was performed using lipofectamine 2000 (Invitrogen, CA, USA). Propidium iodide solution (PI), 3-(4,5-dimethylthiazol-2-yl)-2,5-diphenyltetra-zolium bromide (MTT), rotenone, and 6-OHDA were obtained from Sigma-Aldrich (St. MO, USA). siRNA targeting DX2 (CACGUGCAGGAUUACGGGGC) was purchased from Bioneer (Seoul, Korea).

### Primary neuronal cell isolation

To prepare primary neurons, embryonic brain cortices at E18-E19 were removed and transferred to new conical tubes and gently washed three times with PBS. The washed cortices were incubated in papain solution containing 3.5 mg/ml papain (0.5 units/g; Wako, VA, USA), 0.5 mg/ml EDTA-disodium salt (Wako, VA, USA), and deoxyribonuclease I (DNase I, 5 units/mL; Takara, Shiga, Japan) for 15 min. The digested cortices were dissociated by pipetting 12 times with a glass Pasteur pipette and filtered with a cell strainer (40-mm mesh; BD Biosciences, NJ, USA). The harvested neuronal cells were cultured in MEM containing 20% FBS and N2 supplement (Thermo Fisher Scientific, MA, USA).

### Vialble cell counting

Cells were detached using Trypsin/EDTA solution and subjected to centrifugation at 300 g for 5 min. A portion of the collected cells were then sampled and suspended in a 1:1 mixture of media and trypan blue solution (Cat no. 15250061, Thermo Fisher, CA, USA). The cell suspension was subsequently loaded into a cell counting device (Cat no. AMQAF2001, Countess 3 Autometated cell counter, Thermo Fisher, CA, USA) following the device process. All cell counting experiments were conducted in triplicate for quantification.

### Pulse-chase assay

HEK293 cells transfected with myc-tagged AIMP2 or DX2 were then incubated in a methionine-free medium for 1 h. Then, [^35^S] methionine (50 μCi/ml) was added and incubated for 1 h. After the radioactive methionine was washed off with fresh medium, targeted proteins AIMP2 and DX2 were immunoprecipitated with the myc antibody (SC, CA; USA Cat. #sc-40), separated by 12% SDS-PAGE, and subjected to autoradiography using a BAS scanner (FLA-3000; Tokyo, Japan).

### Primary hippocampal neuron culture and transfection

Hippocampal CA3-CA1 regions from (0–1 day-old) Sprague Dawley rats (DBL; Strain code: NTac:SD) were dissected, dissociated, and plated onto poly-ornithine-coated coverslips inside a 6-mm-diameter cylinder. For live imaging of synaptic physiology, the plasmids (vGlut1-pHluorin with strep-DX2 or strep-AIMP2) were transfected 8 days after plating. Experiments were conducted 14–21 days after plating. All animal experiments were performed with the approval of the Institutional Animal Care and Use Committee of Kyung Hee University. The plasmids were incubated with 2 mM Ca^2+^, 2 × HeBS (273 mM NaCl, 9.5 mM KCl, 1.4 mM Na_2_HPO_4_.7H_2_O, 15 mM D-glucose, 42 mM HEPES), and the mixture was transfected to hippocampal neurons at DIV 8 using the Ca^2+^ phosphate precipitation method.

### Optical live imaging for synaptic function

For synaptic function imaging, we utilized a pHluorin-based assay. Coverslips with neurons were mounted in a laminar-flow-perfusion system of a metal chamber on the stage of a custom-built, laser-illuminated epifluorescence microscope. Live-cell images were acquired using an Andor iXon Ultra 897 (Model #DU-897U-CS0-#BV) back-illuminated EM CCD camera with a diode-pumped OBIS 488 laser (Coherent), shuttered by TTL on/off signal from the EMCCD camera during imaging. Fluorescence excitation/emission and collection were achieved using a 40× Fluor Zeiss objective lens (1.3 NA) with 500–550 nm emission and 498 nm dichroic filters (Chroma) for pHluorin. Action potentials were caused by passing a 1 ms current pulse through platinum-iridium electrodes using an isolated current stimulator (World Precision Instruments). Neurons were perfused in saline-based Tyrode’s buffer consisting of 119 mM NaCl, 2.5 mM KCl, 2 mM CaCl_2_, 2 mM MgCl_2_, 25 mM HEPES, 30 mM glucose, 10 μM 6-cyano-7-nitroquinoxaline-2,3-dione (CNQX), and 50 μM D,L-2-amino-5-phosphonovaleric acid (AP5), adjusted to pH 7.4. All live presynaptic terminal imaging was conducted at 30 °C; all images were acquired at 2 Hz with a 50-ms exposure.

### Immunoprecipitation assay

After washing the prepared cells with cold PBS, cell lysate was collected with 1% Trition × 100 lysis buffer diluted in PBS after incubation at 4 °C for 20 min. The 500 μg of protein in the supernatant was incubated with 1 μg antibody for pull down for 2 h and then incubated with 25 μl protein A/G plus-agarose beads (sc-2003, SC; CA, USA) at 4 °C overnight. After washing the sinked bead with PBS-T (0.05% tween in PBS), 2 × sample buffer was mixed with the bead. For denaturation, the samples were boiled for 10 min. Pull down was performed with PARP-1 antibody, c-Myc antibody for Parkin, and Flag antibody for AIMP2 and DX2.

### Western blot

The prepared protein supernatant was loaded into a 10% acrylamide gel for Western blotting electrophoresis, before transferring the protein from the gel to a PVDF membrane; a 5% skimmed milk solution was then used for blocking. The membrane was incubated in primary antibody diluted with Tris-buffered saline (TBS) with 0.05% Tween (TBS-T) for 2 h. After three washes with TBS-T, the diluted secondary antibody was incubated for 1 h. Detection was performed after luminol (SC; CA, USA). Antibodies against c-myc (sc-40), Ha-tag (sc-7392), PARP-1 (sc-8007), GFP (sc-101525), PCNA (sc-56), and α-tubulin (sc-5286) were purchased from Santa Cruz (SC, CA, USA) and tyrosine hydroxylase antibody (p40101-150) was purchased from Pel-Freez (AR, USA). Poly-(ADP-ribose) (83732s), lamin A/C (2032s), AIF (4642s) and YY1 (46365s) antibodies were purchased from Cell Signalling (MA, USA). For detecting basal AIMP2 and DX2 expression, NMS-01-0011 (curebio, Seoul) was used. All experiments were 3 times repeated independently.

### Ethics statement

All mice experiments were performed under the Kyung Hee University Institutional Animal Care & Use Committee guidelines (KHUASP(SE)-18-101).

### Rotenone-induced PD mouse model

To confirm the anti-neurodegenerative role of DX2 in vivo, chicken-β-actin promoter induced whole body DX2 transgenic animals were provided by Dr. Sunghoon Kim (Seoul National University, South Korea). Male C57Bl/6n-based DX2 transgenic mice and wild type mice were used. Since 8 weeks of age, fresh rotenone solution was orally administrated at a concentration of 30 mg/kg by gavage once a day for 4 weeks to wild type and DX2-TG mice. Rotenone (Sigma-Aldrich, St. Louis, MO, USA) was dissolved in 4% carboxymethylcellulose (CMC, Sigma-Aldrich) with 1.25% chloroform (Beijing Chemical Works, Beijing, China). During rotenone treatment, the rotarod behaviour test was performed once a week, and the latency to fall was measured. At 4 weeks after chemical administration, pole test was performed to measure the turning time above top pole and descending time from top to bottom. To confirm this dopaminergic neuron survival in the substantial nigra, immunohistochemistry staining was performed using tyrosine hydroxylase antibody with cryo-sectioned brain.

### Production and purification of scAAV2

To produce AAV-GFP and AAV-DX2, AAV-GFP and AAV-DX2 were generated by ligating oligonucleotides encoding the GFP or DX2 sequence into the pSF-AAV-ITR-CMV-ITR-KanR (OXGENE, Oxford, UK) vector. AAV viral vectors were prepared and transfected with AAV2 helper (OXGENE, Oxford, UK), AAV2 Rep-Cap (OXGENE, Oxford, UK), and pSF AAV-ITR-DX2 or pSF AAV-ITR-GFP in HEK293 cells. Media and cells were then collected and lysed.

To produce recombinant AAV serotype 2 (rAAV2) encoding DX2 or GFP at 40 L (20 L × 2) scales, one day before transfection of plasmid vectors, HEK 293 cells were counted (8 × 10^5^ cells/ml) and centrifuged at 300 × g for 7 min. The cell pellet was resuspended in HEK293 medium supplemented with 4 mM ultraglutamine and incubated for 24 h. Then, the DNA-PEIproHQ (transfection reagent) complexes (transfection mix) were generated according to the manufacturer’s recommendations. Briefly, two mixtures were generated in culture medium: (1) the plasmid DNA mix (scAAV2 DX2 or GFP–4 mg, rep/cap plasmid–7.2 mg, helper plasmid–8.8 mg, total 20 mg of DNA per 1 L of HEK293 medium with final 4 mM ultraglutamine) and (2) the PEIproHQ mix (60 mg of PEI per 1 L of HEK293 medium with final 4 mM ultraglutamine). These were combined and incubated for 10–15 min at room temperature. Finally, a volume equating to 10% of the production volume was added to each of the wave bags to be transfected (2 L of DNA-PEIproHQ complexes was added to 18 L of culture). Three days post-transfection, the cells were harvested, lysed with lysis buffer containing 300 mM NaCl, 50 mM Tris–Cl, pH8.0, and 0.1% PF68. To remove the residual DNA, the lysate was incubated for 1 h at 37 °C after adding a nuclease. Then, we purified the AAV product using tangential flow filtration (TFF) and affinity chromatography, and the neutralized affinity chromatography eluate material was solubilized in Tris-based buffer solution. In consideration of the effect of the AAV formulation on safety, the Tris-based buffer was changed to PBS-based buffer (PBS with 300 mM NaCl and 0.001% PF-68) by the 2nd TFF diafiltration to produce high-titer virus vector stock. Part of the formulated (diafiltrated) viral particles were concentrated using 100 kDa Amicon Centricon™ and the concentrated products were sterilized by filtration using a 0.22 μm syringe filter (Satorius). A final product (post purification) viral genome (VG) titer of 4.05 × 10^13^ VG (1.62 × 10^13^ VG/mL, by qPCR) was obtained with this process.

### RNA library preparation, sequencing, and data analysis

AAV-GFP or -DX2 was administrated to N2A and SK-N-SH cells. After 3 days, RNA was isolated using TRIzol Reagent (Thermo Fisher Scientific Inc., MA, USA). For control and test RNAs, the construction of the library was performed using QuantSeq 3′ mRNA-Seq Library Prep Kit (Lexogen Inc., VIE, Austria) according to the manufacturer’s instructions. QuantSeq 3′ mRNA-Seq reads were aligned using Bowtie2 [37]. Bowtie2 indices were either generated from genome assembly sequence or the representative transcript sequences for aligning to the genome and transcriptome. The alignment file was used for assembling transcripts, estimating their abundances, and detecting differential expression of genes. Differentially expressed genes were determined based on counts from unique and multiple alignments using coverage in Bedtools [44]. The RC (Read Count) data were processed based on the quantile normalization method using EdgeR within R using Bioconductor [21]. Gene category was based on searches done in DAVID (http://david.abcc.ncifcrf.gov/) and Medline databases.

### Surgical procedures

All experiments in mice were performed under the Kyung Hee University Institutional Animal Care & Use Committee guidelines (KHUASP(SE)-18-101). In the 6-OHDA group, male c57bl/6n mice aged 8 weeks were injected with 4 μg/μl of 6-OHDA in the right striatum. Each mouse was ethically anesthetized using ketamine and a muscle relaxant (Rompun). The anesthetized mice were then placed on the stereotaxic device with tooth and ear bars. The total volume of 6-OHDA per mouse was 3 μl. The injection was performed using a Hamilton syringe at the following coordinates: AP: + 0.5 mm, ML: + 1.8 mm, DV: − 3.7 mm. To evaluate the efficacy of DX2 in the 6-OHDA mice model, AAV-DX2 and its control virus AAV-GFP were injected on both sides of the lesion. The injection was performed using a Hamilton syringe at the following coordinates: coordinates 1: AP: + 1.2 mm, ML: + 1.5 mm, DV: − 3.5 mm; coordinates 2: AP: − 0.1 mm, ML: + 2.1 mm, DV: − 3.2 mm. All stereotaxic injections were performed at a rate of 0.5 μl/min and the needle was removed out of place for another 10 min after the injection before slowly being removed.

### Animal behaviour test in the cylinder

Every mouse in the 6-OHDA model group was behaviourally tested by using a cylinder with a total diameter of 11 cm and height of 20 cm. The blind test method was used through all experiments and analysis. Behaviour tests were conducted at different points after the injection of AAV.

- **Apomorphine-induced rotation test:** Apomorphine-induced rotation test was conducted at 2, 6, and 10 weeks post-injection of 6-OHDA. To avoid possible hypersensitization, an interval of a minimum of 3 to 4 weeks was chosen. Each mouse received 0.5 mg/kg of apomorphine hydrochloride via subcutaneous injection (Sigma-Aldrich) and then was placed in the individual cylinder described above. Mice were allowed to adjust to their environment for 5–10 min before any turns ipsilateral or contralateral to the lesion site. All procedures were recorded for 35 min. Results were organized by net contralateral rotations per minute.
- **Cylinder test:** Forelimb movement was analyzed by the cylinder test using a modified mouse version [47]. Every mouse in the 6-OHDA group was tested using a cylinder (11 cm in diameter and 20 cm in length). All analyses were performed by an experimenter blinded to the group. Behaviour tests were conducted at different points after the AAV injection. The cylinder test was conducted at 2, 6, and 10 weeks post-injection of 6-OHDA at the onset of the dark cycle. Each mouse was placed in the plastic cylinder described above.
- **Elevated body swing (EBS) test:** The EBS test was performed after the cylinder test in the 6-OHDA model. Each mouse was held 3 cm vertically above the ground for 60 s. The swinging pattern of each mouse was recorded and marked whenever the mouse turned its head over 30 degrees from the axis. Each test was conducted with two examiners: one was holding the mouse while the other recorded the movement. Each test was recorded as a ratio of turns to the total number of swings.

### Pole test

The mouse was placed on top of a pole, and the duration for which the mouse stayed on the pole was measured. Since mice with deficits in motor activity will likely fall off the pole and mice with improved motor activity will descend the pole, an increase in the time in pole descent indicates an improvement in motor activity

### Rotarod test

To examine the motility of the rotenone mouse models, a rotarod test was conducted at 3 and 15 days post lesion. All mice were trained on the same machine with equal rotating speed to show a stable performance. Training procedures consisted of three sessions on 2 days in which each session included three individual trials, lasting at least 200 s each. All mice were trained at 5, 10, and 15 rpm (revolutions per minute). The final test was conducted at 15 rpm (three sessions, 300 s each) on the third day. To reduce fatigue, each mouse was given at least 5 min of rest between each measurement.

### Tissue preparation

At the end of the behavioural assessment, the total duration was 12 weeks for the 6-OHDA model. Mice were sacrificed after being anesthetized with isoflurane and perfused with 0.9% saline mixed with 4% paraformaldehyde (PFA; Yakuri Pure Chemicals) in phosphate buffer (PBS) at a pH of 7.4. Organs including the lungs, heart, liver, kidney, ovary, spleen, bladder, spinal cord, and brain were collected. The brains were fixed overnight in 4% PFA followed by a dehydration step with 30% sucrose in PBS. Brain samples were then frozen with optimum cutting temperature compound (O.C.T. compound; Tissue-Tek) and cut on a cryotome (Leica CM1850; Leica Biosystems) into 30 μm thick coronal sections through the entire substantia nigra and striatum. All sections were made from the posterior end of the SN to the anterior end of the striatum. The sections were stored at − 20 °C in a cryoprotectant solution made with ethylene glycol, glycerol, and 0.2 M phosphate buffer.

### Immunofluorescence staining

For the staining, the floating brain tissues in cryoprotectant solution or attached cells on a coverslip were moved to a slide glass. After washing with PBS three times for 5 min, blocking with 5% BSA in PBS was performed for 30 min followed by permeabilization with 0.2% triton × 100 in PBS. The primary tyrosine hydroxylase antibody was 1:500 diluted with 1% BSA in PBS, applied to the section/cells, and incubated at room temperature for 1 h. After three washes with PBS-T (0.1% tween in PBS) for 5 min, the secondary antibody diluted with 1% BSA in PBS was incubated in a dark room for 1 h. Mounting with VECTASHILD antifade mounting medium with DAPI was used for nucleus counterstaining.

### Image analysis

Images were analyzed with Image J (http://rsb.info.nih.gov/ij) using the Time Series Analyzer plugin, accessible at https://imagej.nih.gov/ij/plugins/time-series.html. Synaptic boutons of neurons were appointed as oval regions of interest (diameter, 10 pixels), and the amplitude of pHluorin-based fluorescence at synapses was counted using Origin Pro 2020. The kinetics of endocytosis was measured using a single exponential decay function.

### Statistics analyses

The *p* values are represented for mean ± SEM and data were statistically analyzed using Student's *t*-test or ANOVA, where appropriate. A *p*-value of less than 0.05 was considered to be statistically significant.

## Results

### Differential binding affinities of AIMP2 and DX2 to Parkin and PARP-1

**Fig. 1:**
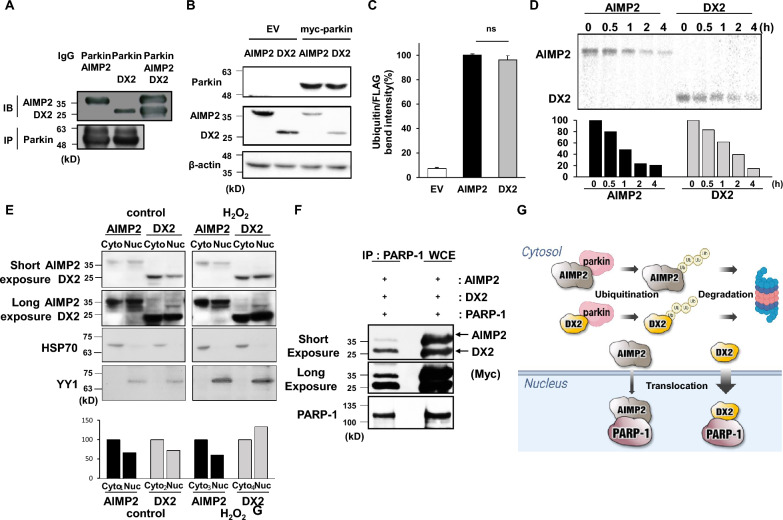
Binding affinity of AIMP2 and DX2 to PARKIN and PARP-1 **A** Myc-tagged AIMP2 and DX2 DNA plasmid were co-transfected with Flag-parkin in Hek293 cell line. After 24 h, the cells were harvested and cell lysates were immunoprecipitated by flag-antibody. **B** Flag-tagged AIMP2 and DX2 were transfected with empty vector (EV) or myc-tagged parkin. The expression level of AIMP2 and DX2 were decreased after parkin tansfection at a similar level. **C** A graph quantifying the ubiquitination of AIMP2 and DX2 by PARKIN. (ns, non- significant) qPCR was performed in triplicate. **D** AIMP2 and DX2 protein stability were assessed with cycloheximide-treated pulse chase assay. The samples were harvested in a time-dependent manner at 0–4 h and examined using immunoblot assays. The quantification was measured by image J (lower bar graph). **E** Myc-tagged AIMP2 and DX2 transfected samples were collected separately from cytosol and nucleus. HSP70 and YY1 protein were used as fractionation markers. **F** AIMP2 and DX2 expression was induced by transfection of each plasmid in SH-SY5Y cells followed by analyses with PARP-1 pull-down assays; DX2 had much higher binding affinity with PARP-1 than AIMP2. (WCE; whole cell extract) **G** Schematic Fig. about the fate of AIMP2 and DX2. Both AIMP2 and DX2 are substrates for Parkin in the cytosol, and DX2 translocates to the nucleus more easily than AIMP2. **(**ns, non-significant)

In our previous study, we demonstrated that while AIMP2 binds to TRAF2 and p53, inducing apoptosis, DX2 counteracts the apoptotic function of AIMP2 by obstructing its binding to TRAF2 and p53, thereby masking the interaction site of AIMP2 [13]. Moreover, it has been reported that wild-type Parkin1 interacts with AIMP2, resulting in its degradation. However, mutant Parkin1 fails to bind AIMP2, which is one of the molecular mechanisms underlying mutant Parkin1-induced parkinsonism. Interestingly, DX2 displayed a comparable binding affinity to AIMP2 for Parkin1 interaction (Fig. 1a), and both AIMP2 and DX2, as Parkin substrates, were degraded at similar levels (Fig. 1b). Additionally, under conditions treated with MG132 proteasome inhibitor, parkin not only ubiquitinated AIMP2 but also DX2 similarly (Fig. 1c and Additional file 1: Fig. S1). Considering that protein stability may affect binding opportunities to a target molecule, we compared the stabilities of AIMP2 and DX2. The pulse-chase assay revealed that DX2 maintained a similar stability to full-length AIMP2 for up to 4 h after halting de novo protein synthesis with cycloheximide (Fig. 1d). AIMP2 and DX2 are known to translocate into the nucleus in response to cellular stress [39]. Under hydrogen peroxide (H_2_O_2_)-treated conditions, DX2 appeared to localize in the nucleus more rapidly than AIMP2 (Fig. 1e). It has been previously demonstrated that AIMP2 interacts with PARP-1, leading to aberrant neuronal cell death in PD [38]. Consequently, we investigated whether DX2 also binds to PARP-1. In a pull-down assay with PARP-1, the binding affinity of DX2 to PARP-1 was found to be stronger than that of AIMP2 (Fig. 1f), suggesting that DX2 has the potential to modulate neuronal cell fate in contrast to AIMP2 during PARP-1 activation.

Taken together, our results show that both AIMP2 and DX2 can be ubiquitinated and degraded in the cytoplasm as common substrates of Parkin (upper panel of Fig. 1g). However, DX2 responds to reactive oxygen species (ROS) signals and predominantly localizes in the nucleus, potentially having more opportunities to bind to PARP-1 (lower panel of Fig. 1g).

### DX2 modulates AIMP2-PARP-1 interaction and inhibits PARP-1 activation

**Fig. 2:**
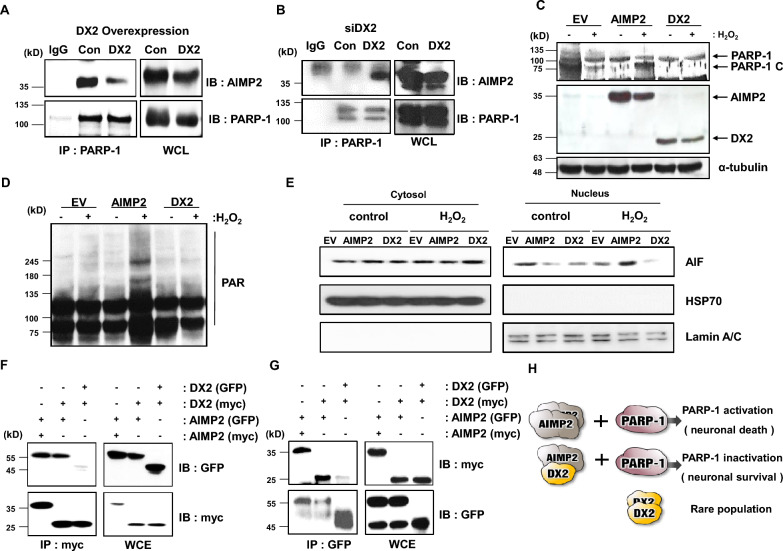
Bi-phasic effect of AIMP2 and DX2 on PARP-1 activity and parthanatos **A** After DX2 overexpression, binding between AIMP2 and PARP-1 was determined by Western blotting in SH-SY5Y cells. **B** DX2 siRNA (DX2) and control siRNA (Con) were transfected into SH-SY5Y cells and incubated for 48 h. Total cell lysates were incubated with protein agarose beads to immunoprecipitate PARP-1 bound AIMP2, which is then analyzed by immunoblot analysis. **C** and **D** Cells were transfected with the EV (empty vector), AIMP2, and DX2; after 24 h, transfected cells were incubated with 100 μM H_2_O_2_ for 24 h. Cleaved PARP-1 levels **C** and PARylation **D** are shown. **E** Flag-tagged EV, AIMP2 and DX2 was transfected in SH-SY5Y cell line. Transfected cells were incubated with 200 μM H_2_O_2_ for 24 h. HSP70 antibody was used for cytosol marker and Lamin A/C antibody was used for nucleus marker. **F** and **G** GFP- or myc-tagged AIMP2 and/or DX2 expressing plasmid were transfected into cells and binding affinity was measured by immunoprecipitation (IP) with a myc antibody **F** and GFP antibody (**G**). **H** Schematic Fig. of AIMP2-induced PARP-1 activation. In the absence of DX2, AIMP2 dimers induce PARP-1 activation and neuronal death. However, in the presence of DX2, DX2 interacts with AIMP2 and inhibits PARP-1 activation

To determine whether DX2 acts as a competitive inhibitor of AIMP2, suppressing AIMP2-induced PARP-1 activation and neuronal cell death, we transfected neuroblastoma cells with DX2-plasmid vectors and -siRNA, and subsequently measured the binding of AIMP2 to PARP-1. We found that AIMP2-PARP-1 binding was reduced in DX2-overexpressing cells (Fig. 2a) and increased in DX2-knockdown cells (Fig. 2b; Additional file 1: Fig. S2), indicating that DX2 expression levels play a critical role in AIMP2-PARP-1 interactions. Given that PARP-1 activation occurs upon oxidative stress and its cleavage serves as a marker for apoptosis, we monitored PARP-1 cleavage in cells transfected with empty vector (EV), AIMP2, or DX2, both in the absence and presence of hydrogen peroxide (H_2_O_2_). AIMP2-transfected cells exhibited increased PARP-1 cleavage under oxidative stress conditions compared to other transfected cells (Fig. 2c, fourth lane). Notably, PARP-1 cleavage was not observed in DX2-transfected cells (Fig. 2c). Since PARylation is a post-translational process indicative of PARP-1 activation, we investigated the effects of AIMP2 or DX2 on PARylation. AIMP2 enhanced PARylation in the presence of H_2_O_2_, whereas DX2 had no effect (Fig. 2d). Next, to determine whether the PARylation are actually involved in parthanatos, we examined the nucleus translocation of AIF, one of the markers of parthanatos under H_2_O_2_-treatmed condition. Expectedly AIMP2 overexpression induced more nucleus localization of AIF and reversely DX2 seemed to suppress the translocation of AIF (Fig. 2e). Considering that AIMP2 tends to form dimers [42], we sought to determine if AIMP2-DX2 heterodimers exist and whether homo- or heterodimer formation impacts PARP-1 activation. To assess the binding affinities of AIMP2 and DX2 and the likelihood of heterodimer formation, we employed two different tags, green fluorescent protein (GFP) and myc, on each gene. Pull-down assays with myc-tag on transfected cell lysates revealed the formation of AIMP2 homodimers and AIMP2-DX2 heterodimers (Fig. 2f, first and second lanes of left upper panel). However, the amount of DX2 homodimers was considerably lower (Fig. 2f, third lane of upper left panel) compared to AIMP2-AIMP2 homodimers or AIMP2-DX2 heterodimers. Immunoprecipitation with GFP tag yielded a similar binding pattern between AIMP2 and DX2 as observed in Fig. 1K when immunoprecipitated by myc tag in the opposite direction (Fig. 2g).

Collectively, our findings suggest that when DX2 is insufficiently expressed, AIMP2 forms homodimers, interacts with PARP-1, activates PARP-1, and induces neuronal cell death. However, as DX2 exhibits a significantly higher binding affinity for PARP-1 than AIMP2, DX2-expressing cells display reduced AIMP2-PARP-1 binding, leading to the inhibition of PARP-1 activity and neuronal survival. These results indicate that DX2 is a crucial factor for neuronal cell viability under PARP-1 activating conditions, and that DX2 overexpression may ameliorate neuronal cell death in neurodegenerative diseases (Fig. 2h).

### DX2 overexpression mitigates AIMP2-induced cell death and protects synaptic functionality in neuronal cells

**Fig. 3:**
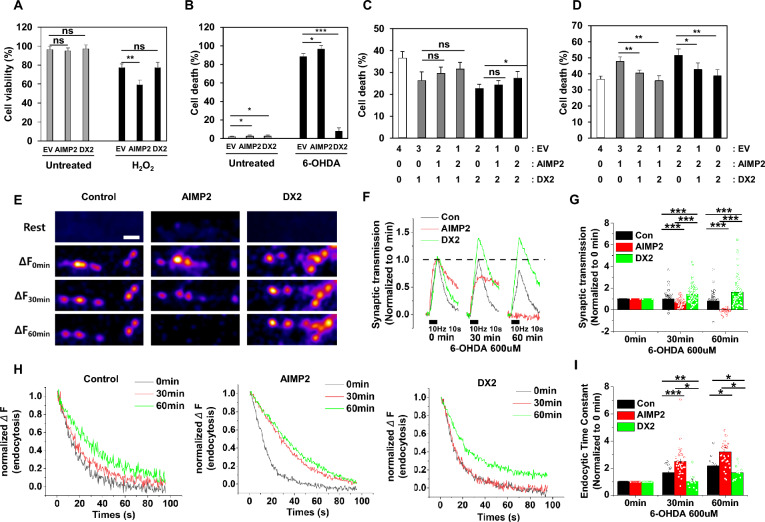
DX2 reduced neuronal apoptosis **A** SH-SY5Y cells were transfected with EV, AIMP2, and DX2 expression plasmids, incubated with 200 μM H_2_O_2_ for 24 h, and cell viability was measured with trpyphan blue-based cell counting device. **B** SH-SY5Y were transfected with EV, AIMP2, and DX2 expression plasmids and incubated with or without and treated with 100 μM 6-OHDA for 24 h. The cell death was analyzed by Propidium Iodide (PI) staining. **C** and **D** SH-SY5Y cells are co-transfected with EV, AIMP2, and DX2 expression plasmids, incubated with 200 μM H_2_O_2_ for 24 h, and cell viability was measured with cell counting device. The number shows the amounts of transfected DNA. **E** Representative images of the vGlut-pH response at synapses in Control, AIMP2-overexpressing, and DX2-overexpressing neurons. Neurons transfected with vG-pH or vG-pH with AIMP2 or vG-pH with DX2 were stimulated with 100 Action Potentials (APs) at 10 Hz in the presence of 6-OHDA (600 μM) for 0, 30, and 60 min. The scale bar: 5 μm. **F** Average traces of vGlut-pH in the response of 100 APs in Control (black), AIMP2- (red), and DX2-overexpressing neurons (green) in the time course of 6-OHDA incubation as described. The traces were normalized to the peak of trace at 0 min. **G** The mean values of the peak of synaptic transmission in Control (black), AIMP2- (red), and DX2-expressing (green) neurons in the time course of 6-OHDA incubation. [Control]_30 min_ = 0.99 ± 0.06 (n = 83 boutons), [AIMP2]_30 min_ = 0.68 ± 0.05 (n = 46 boutons), [DX2]_30 min_ = 1.47 ± 0.12 (n = 55 boutons); [Control]_60 min_ = 0.81 ± 0.06 (n = 83 boutons), [AIMP2]_60 min_ = − 0.04 ± 0.03 (n = 46 boutons), [DX2]_60 min_ = 1.63 ± 0.19 (n = 55 boutons) (***p < 0.001, **p < 0.01, *p < 0.05). Data were expressed as mean ± SEM. **H** Average traces of vG-pH endocytosis after stimulation of 100 APs in Control (left), AIMP2- (middle), and DX2-overexpressing (right) neurons in the presence of 6-OHDA for 0, 30, and 60 min. **I** The normalized mean values of post-stimulus endocytic time constants in Control, AIMP2-, and DX2-overexpressing neurons in the course of 6-OHDA incubation for 0, 30, and 60 min. [Con]τ_endo30min_ = 1.67 ± 0.14 (n = 14 boutons), [AIMP2]τ_endo30min_ = 2.50 ± 0.20 (n = 32 boutons), [DX2]τ_endo30min_ = 1.04 ± 0.15 (n = 12 boutons); [Con]τ_endo60min_ = 2.17 ± 0.17 (n = 14 boutons), [AIMP2]τ_endo60min_ = 3.22 ± 0.29 (n = 32 boutons), [DX2]τ_endo60min_ = 1.68 ± 0.15 (n = 12 boutons). (***p < 0.001, **p < 0.01, *p < 0.05). Data were expressed as means ± SEM. Each endocytosis was normalized to 0 min endocytosis time constant

Drawing upon these findings, we propose that DX2 acts as an inhibitory molecule in oxidative stress-induced PARP-1 activation. We investigated the influence of AIMP2 and DX2 on cell death in neuronal cells. Plasmids expressing either AIMP2 or DX2 were introduced into SH-SY5Y neuroblastoma cells. Subsequently, apoptosis was induced through ROS stress by treating the cells with H_2_O_2_. Under normal conditions, overexpression of AIMP2 or DX2 had little impact on cell viability; however, in H_2_O_2_-treated conditions, AIMP2 overexpression significantly decreased cell viability, while DX2 overexpression mitigated this effect (Fig. 3a). When cells were treated with 6-OHDA, a selective neurotoxin targeting dopaminergic neurons, AIMP2 administration induced cell death in a statistically significant manner compared to untreated cells. Intriguingly, DX2 more potently suppressed cell death relative to 6-OHDA-treated conditions (Fig. 3b).

Subsequently, we examined the role of AIMP2 and DX2 in regulating cell viability under H_2_O_2_-induced cell death conditions. Even with increased AIMP2 expression (1–2 μg), a 1 μg transfection of DX2 diminished AIMP2/ H_2_O_2_-induced cell death to a level comparable to that achieved with 2 μg of DX2 (Fig. 3c). Conversely, AIMP2 expression levels had no significant effect on neuronal cell death in DX2-expressing cellular conditions (1–2 μg) (Fig. 3d). Consequently, DX2-expressing cells exhibited substantially reduced cell death compared to control cells under oxidative stress conditions. Collectively, these findings indicate that the anti-apoptotic effect of DX2 is considerably more potent than the pro-apoptotic activity of AIMP2 when both proteins are co-expressed.

We hypothesized that DX2 expression confers a protective effect on synaptic functionality in a neurodegenerative model system. To validate this notion, we employed a pHluorin-based assay, which is widely used for monitoring synaptic functionality, including synaptic transmission and synaptic vesicle endocytosis [2, 34]. Primary hippocampal neurons were transfected with vGlut-pHluorin in combination with either AIMP2 or DX2, and activity-driven synaptic transmission was monitored in the presence of 6-OHDA. Treatment with 6-OHDA resulted in a significant decrease in synaptic transmission to 80% at 60 min compared to control neurons, with AIMP2-overexpressing neurons exhibiting an even greater reduction in synaptic transmission, as evidenced by a loss of bouton signal (Fig. 3e–g). In contrast, DX2-overexpressing neurons demonstrated no deficits in synaptic transmission and, in fact, enhanced synaptic function. To evaluate endocytic activity, which reflects neuronal damage, we indirectly measured endocytosis by monitoring changes in the exocytic signal using the declining curve from the peak. In synaptic vesicle retrieval, AIMP2-overexpressing conditions displayed lower activity than control conditions, while DX2-overexpressing neurons exhibited a clear protective effect in the presence of 6-OHDA (Fig. 3h and i; for example, compare with the y-axis value of the red curve at 30 s), suggesting that DX2 promotes neuronal survival in neurotoxic environments.

### AAV-DX2 treatment reduces cell death and modulates survival gene expression, suggesting therapeutic potential.

**Fig. 4:**
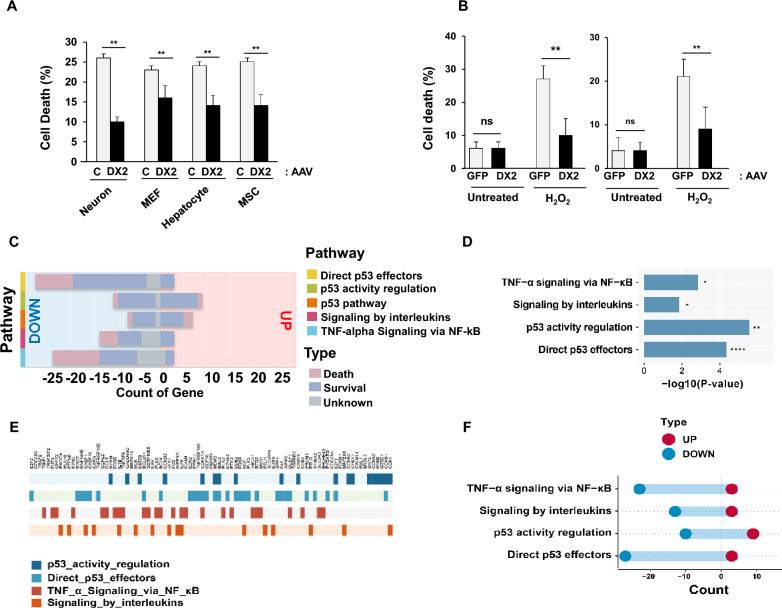
AAV-DX2 has anti-apoptotic effects and alters cellular signalling. **A** Cytotoxic effects in the primary neurons (Neuron), MEF (Mouse embryonic fibroblasts), hepatocytes, and MSC (Mesenchymal stem cells). After H_2_O_2_ (200 μM, 24 h) treatment, a decreased cytotoxic effect was observed in DX2 transduced cells (DX2) when compared with control-transduced cells. The measurement of cell death was conducted through cell counting. **B** DX2 is not required for normal cell growth in SH-SY5Y (left) and primary neuronal cells (right). In oxidative stress conditions, DX2-infected cells (AAV-DX2, DX2) showed decreased levels of cell death when compared to their control counterparts (AAV-GFP, GFP). The measurement of cell death was again performed using cell counting. **C** Graph of gene counts representing number of survival-related and death-related genes in each pathway. **D** *P*-value plot of the signalling pathway changed by DX2 overexpression **E** Enrichment plot of RNAseq of AAV-GFP or AAV DX2-infected neuroblastoma cell. **F** Graph of gene counts representing cell death and inflammatory related pathways were downregulated.; *ns* Non-significant; *P < 0.05; **P < 0.01; ****P < 0.0001 (*t*-test)

To assess whether AAV-DX2 treatment exhibits the same anti-apoptotic properties as DX2 overexpression, we exposed a variety of cell types, including primary neurons, mouse embryonic fibroblasts (MEF), hepatocytes, mesenchymal stem cells (MSC), and neuroblastoma cells to AAV-DX2. Notably, AAV-DX2-transduced cells consistently demonstrated a marked reduction in cell death compared to AAV-GFP-transduced cells following H_2_O_2_ treatment (Fig. 4a), suggesting that AAV-DX2 serves as an effective anti-apoptotic intervention. Intriguingly, under basal conditions, cell viability remained unaffected by the presence or absence of AAV-DX2, indicating that DX2 operates exclusively in response to stress-induced apoptotic stimuli (Fig. 4b).To elucidate the therapeutic mode of action and anticipate potential unexpected responses, we treated SK-N-SH human neuroblastoma cells with AAV-DX2 and performed RNA-seq analysis. Differentially expressed genes (DEGs) were classified into signaling pathways or ontology databases, revealing a common involvement in suppressing p53-associated cell death pathways and TNF-α or interleukin-related signaling (Fig. 4c). These findings corroborate previous knowledge of AIMP2 and DX2-related pathways and display a predominance of downregulated genes (Fig. 4d). By characterizing each gene, we aimed to understand its contribution to cell death or survival (Fig. 4e, Additional file 1: Table S1). Statistical significance was visualized through a p-value distribution graph for all pathways following DX2 overexpression (Fig. 4f).

In summary, AAV-DX2-based gene therapy is anticipated to downregulate inflammation or oxidative stress-induced neuronal apoptosis-related genes while upregulating neurosurvival-associated genes, highlighting its potential as a promising therapeutic approach.

### DX2 overexpression in transgenic mice confers resistance to parkinsonism induced by rotenone treatment

**Fig. 5:**
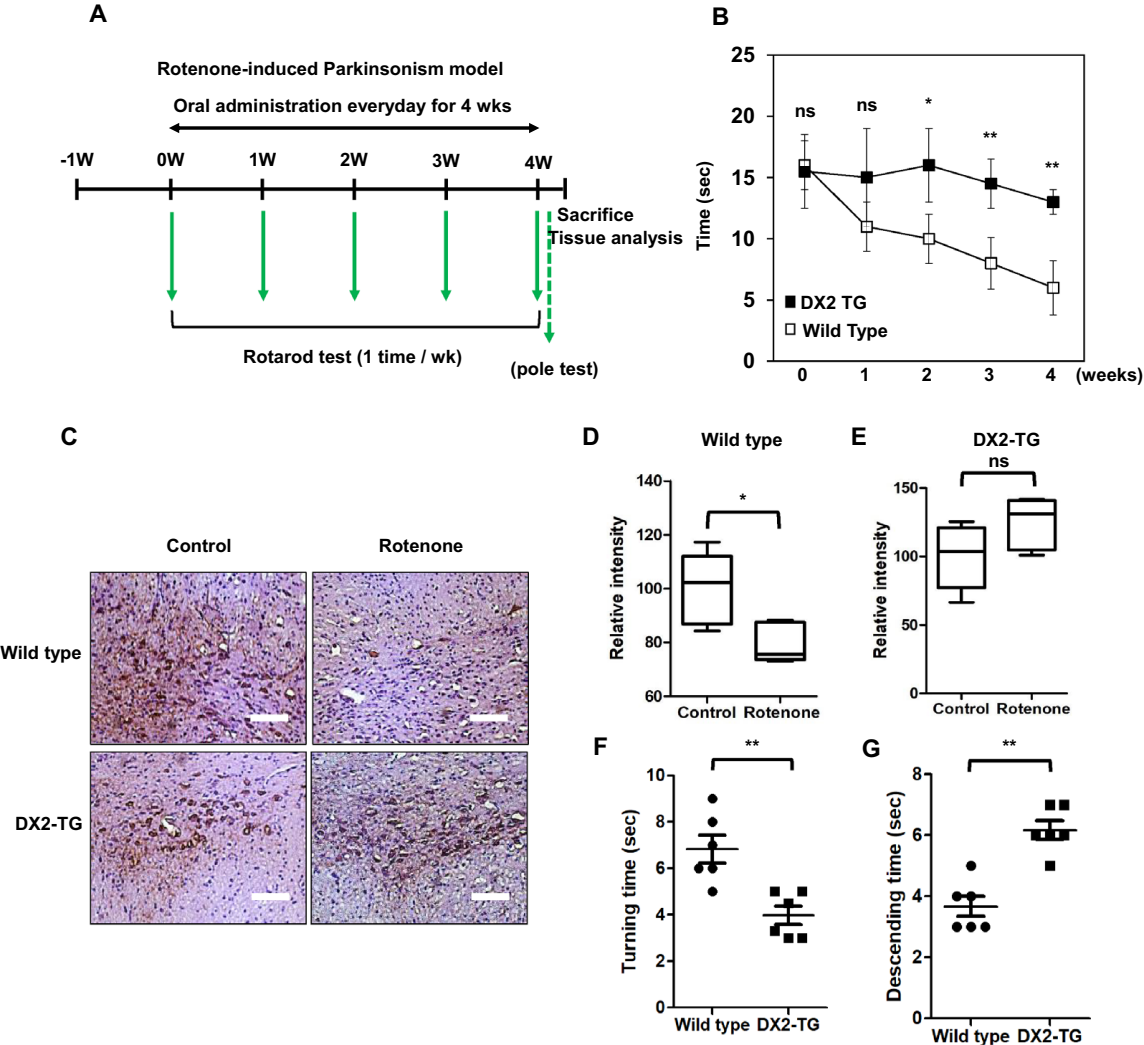
DX2 transgenic mice show neuroprotective effect **A** Schedule of rotenone-based PD induction in DX2 transgenic mice. **B** Rotarod analysis. Latency to fall in rotenone-treated wild type and DX2 transgenic (TG) mice. **C** TH expression was analyzed in the substantia nigra region of indicated mice. The scale bar: 50 μm. **D** and **E** Relative TH-positive staining intensity between control and rotenone-treated wild-type animals. **E** Relative TH-positive staining intensity between control and rotenone-treated DX2-TG animals. **F** and **G** The Pole test. Vertical movement and T-turn time in rotenone-treated wild type and DX2 TG mice. Animals: *n* = 6 (in each group); *ns* non-significant; *P < 0.05; **P < 0.01; ***P < 0.001

To determine the importance of DX2 expression in Parkinson's disease (PD) treatment, we generated whole-body DX2-overexpressing transgenic mice (DX2-TG). Additionally, there is are no different life span between WT and DX2-TG mice (Additional file 1: Fig. S3) We administered rotenone to both control and DX2-TG mice to examine its effects on parkinsonism. Four weeks after rotenone treatment, we conducted rotarod and pole tests and analyzed the mice's behavior (Fig. 5a). We observed a reduction in motor coordination in rotenone-treated wild-type mice; however, the rotenone-treated DX2-TG mice demonstrated significant resistance to the decline in motor activity (Fig. 5b). Additionally, we assessed the expression of tyrosine hydroxylase (TH), a marker for dopaminergic neurons. Under normal conditions, there was no difference in TH expression between wild-type and DX2-TG mice, implying normal viability of dopaminergic cells in the substantia nigra pars compacta (SNpc). In contrast, under rotenone-treated conditions, TH expression was significantly reduced in wild-type mice, while TH-positive cells in DX2-TG mice displayed notable recovery compared to cells from wild-type mice (Fig. 5c–e). We also confirmed improvements in motor symptoms in DX2-overexpressing mice following rotenone treatment. In the pole test, DX2-TG mice turned around faster than wild-type mice (Fig. 5f) and exhibited a longer descending time, indicative of a stronger grip on the pole (Fig. 5g). In conclusion, our results reveal that DX2 overexpression in transgenic mice confers resistance to Parkinsonism induced by rotenone treatment, suggesting a potential therapeutic role for DX2 in PD.

### Neuroprotective role of AAV-DX2 in the 6-OHDA-induced PD model

**Fig. 6:**
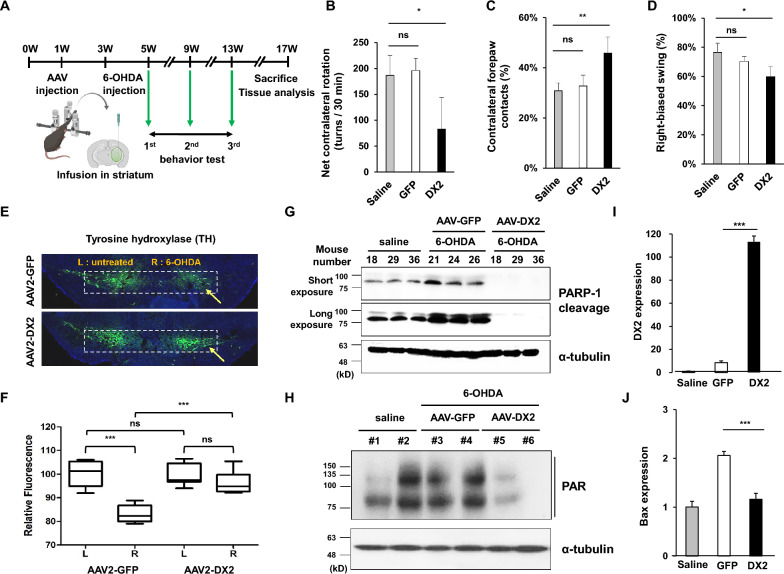
DX2 prevents behavioural deficits in the 6-OHDA -induced PD model **A** Scheme of AAV-DX2 transduction in the 6-OHDA-induced mouse model. **B** AAV-DX2-treated mice showed lower levels of contralateral rotation compared to saline or vehicle (GFP), indicating that DX2 attenuated damage in dopaminergic neurons. **C** DX2-treated mice showed increased contralateral forepaw contacts, indicating that AAV-DX2 attenuated unilateral damage in dopaminergic neurons. **D** AAV-DX2 treated mice show less right-biased body swing. Animals: saline (saline-treated wild type mice), n = 4; GFP (GFP-injected 6-OHDA-treated mice), n = 5; DX2 (DX2-injected 6-OHDA-treated mice), n = 11. AAV: AAV-GFP, 4 × 10^9^ vg; AAV-DX2, 4 × 10^9^ vg. **E** Immunofluorescence image of the GFP and DX2-injected mouse brain. The white square box indicates TH-positive dopaminergic neuronal cells and the yellow arrows show the virus injection site. The relative fluoresence. **F** Relative fluoresence between AAV-GFP and AAV-DX2 group. Quantification of fluoresence intensity was conducted using the Image J software. L: Left. R: Right. **G** Immunoblot assay of the GFP and DX2 injected mice. The cleaved PARP-1 specific antibody was used. Which lanes were randomly selected mice in each group. **H** Immunoblot assay about the samples from AAV-GFP or DX2-injected mice under 6-OHDA treated comdition. The parylation-specific antibody was used. Which lanes were randomly selected mice in each group. **I** and **J** DX2 and Bax mRNA expression of naïve, 6-OHDA, and DX2-treated mice. *ns* Non-significant; *P < 0.05; **P < 0.01; ***P < 0.001

To investigate the effects of DX2 in the 6-hydroxydopamine (6-OHDA)-induced Parkinson's disease (PD) model [27], mice were first intracranially injected with either AAV-GFP (control) or AAV-DX2 and subsequently received a 12 μg injection of 6-OHDA in the right striatum. Two weeks later, mouse behavior was assessed twice at 4-week intervals (Fig. 6a). According to the apomorphine test, motor symptoms were notably ameliorated in DX2-treated mice compared to saline or GFP-treated mice (Fig. 6b).

Furthermore, to compare the neuroprotective effects between AAV-GFP and AAV-DX2-treated mice, we conducted the contralateral forepaw contact test and the elevated body swing test. In the cylinder test, both saline and GFP mice exhibited unequal distribution of touch. However, the touch rate of contralateral and ipsilateral paws increased in the AAV-DX2-injected group (Fig. 6c). In the elevated body swing (EBS) test, head swing to the right was diminished in the AAV-DX2-injected group (Fig. 6d). Collectively, mouse behavior was indistinguishable between saline and GFP-treated mice, yet recovery of 6-OHDA-induced PD symptoms was observed in AAV-DX2-treated mice.

To further analyze tyrosine hydroxylase (TH) expression in the AAV-transduced mouse brain, we performed immunostaining. The 6-OHDA-injected right hemisphere displayed a decrease in TH intensity due to the loss of dopaminergic neurons in the substantia nigra (Fig. 6e and Additional file 1: Fig. S4). However, the number of dopaminergic neuronal cells recovered in DX2-injected mice compared to GFP-transduced ones (Fig. 6e and f).

We assessed the cleavage level of poly (ADP-ribose) polymerase 1 (PARP-1), a marker of apoptosis, in the striatum region of the tested animals. Cleavage of PARP-1 was little detected in the striatum of AAV-DX2-injected mice even after 6-OHDA injection (Fig. 6g). Also, PARP-1 was deactivated in the striatum region of AAV-DX2-injected mice compared with 0.9% saline or AAV-GFP-injected mice (Fig. 6h). To confirm the anti-apoptotic effect on dopaminergic neurons in DX2-injected mice, we analyzed the mRNA expression levels of DX2 and the apoptotic marker gene, Bax, using quantitative RT-PCR. As anticipated, DX2 expression levels significantly increased in DX2-injected mice (Fig. 6i), and Bax expression, a neuronal cell death marker, diminished in these mice (Fig. 6j).

In conclusion, our findings demonstrate that AAV-DX2 administration improves motor function and neuroprotection in a 6-OHDA-induced PD mouse model. This study underscores the therapeutic potential of DX2 for PD treatment and emphasizes the need for further research to optimize and develop this approach for clinical applications.

## Discussion

The most important finding of this study was that DX2, a splice variant of AIMP2 [11], can effectively turn off PARP-1 activation. The DX2 transgene encoded by rAAV2 vector system, competed with AIMP2, bound to PARP-1 more strongly than AIMP2, but did not induce its overactivation, resulting in neuronal cell survival. Over the past decade, PARP-1 inhibitors have been explored in a few clinical trials to treat ischemia [51]. However, it still has a technical limitation in effectively accessing the central nervous system. Since PARP-1 activation is critical for sensing and recovery of DNA damage [56], systemic or long-term treatment with PARP-1 inhibitors may cause serious side effects, like teratogenicity and anaemia [36, 57].Thus, spatiotemporal or conditional inhibition would be preferred in clinical interests. DX2, a competitive antagonist of AIMP2, could be an alternative for inhibiting the PARP overactivation pathway, since AIMP2 is an upstream regulator of PARP-1 in the cell death regulation and AIMP2 accumulation results in PARP-1 overactivation and cell death, even without DNA damage [38].

Thus, using DX2 for selective inhibition of PARP-1 overactivation, only when AIMP2 accumulation is present, would be a smart strategy for treating PD effectively while avoiding unnecessary adverse effects due to the use of PARP inhibitors.

Chronic diseases, such as PD, need long-term intervention. Many allopathic medications are applicable for patients with PD, but all of them have considerable disadvantages in controlling the symptoms. Levodopa, one of the representative drugs for the treatment of PD [41, 43], has many side effects, such as the end-of-dose deterioration of function or on/off oscillations, especially in patients on chronic levodopa therapy [8, 16]. To overcome the side effects of levodopa, many other drugs, including dopamine agonists, MAO-B inhibitors [25, 28], and COMT inhibitors [6, 54] are used; however, they also have other side effects. To treat patients with PD more efficiently, novel drug targets with fewer side effects than conventional drugs need to be developed; ideally, the new drug should assist the survival of dopaminergic neurons [24], besides dopamine production or secretion. In this study, DX2 was shown to improve motor activity (Figs. 4, 7 and 8) and rescue dopaminergic neuronal cell death (Figs. 7e and 8e).

Parkin is frequently mutated or inactivated in patients with sporadic and familial PD [1, 32]. In previous reports, AIMP2 had been reported as a pro-apoptotic protein ubiquitinated by Parkin E3 ubiquitin ligase, and to be highly expressed in patients with PD [38]. Malfunctional PD can cause AIMP2 protein to accumulate in cell, leading to aberrant cell death. A portion of the alleviated AIMP2 seems to be located in the nucleus to bind to PARP-1 [38], hence inducing parthanatos. DX2, lacking exon 2, functions as a competitive antagonist of AIMP2 [12, 23]. However, since DX2 seems to act only when extra amount of AIMP2 is present, induced in a specific stress condition (Figs. 1, 2 and 3), logically its overexpression is non-oncogenic and it barely disturbs the tumour suppressive function of AIMP2 in normal condition (Fig. 6b and Additional file 1: Fig. S5) [10].

Although majority of our study confirmed the preventive effect of DX2 on 6-OHDA-induced mouse model, the concept of prevention and therapy in the PD-induced mouse can be used interchangeably. Even though we tested a couple of preventive concept animal models, the results indicated remarkable therapeutic application prospects. Neurodegeneration is an event caused by imbalance in the neuronal population undergoing survival and death. If, with AAV-DX2 therapy, we can increase the surviving cell population and suppress cell death, the rate of natural neuronal regeneration may be increased [35]; delay of neurodegeneration may occur via natural neuronal regeneration [31]. Therefore, improved motor activity and reduced dopaminergic neuronal cell death due to DX2 overexpression in the substantia nigra of PD mouse model are important observations that could be exploited as therapeutic targets. In the 6-OHDA-induced Parkinson’s disease model, since 6-OHDA does not cross the blood–brain barrier, it needs to be injected directly into the substantia nigra, medial forebrain bundle, or striatum to inhibit the nigrostriatal dopaminergic pathway [29]. It has been reported that 6-OHDA acts specifically on monoaminergic neurons through increased ROS and quinone [20, 46], and the formation of Lewy body is not induced in such mice [55]. Chronic administration of rotenone causes nigrostriatal dopaminergic degeneration and Parkinson's symptoms while increasing the α-synuclein and significantly increasing Lewy body inclusions [5]. In particular, inhibition of systemic complex I by rotenone is known to induce selective effects of oxidative stress on the nigrostriatal dopamine system compared to that in the MPTP model [48]. Since none of the mouse models of Parkinsonism, induced by a neurotoxin, could explain PD symptoms completely, we confirmed that the chemicals effectively induced aberrant activation of PARP-1. DX2 seemed to be an effective therapeutic tool for promoting the survival of dopaminergic neuronal cells in all the three mouse models discussed above. Further, we showed the expression level of DX2 to be important for the prevention of progressive motor deficits after neuronal damage. Collectively, we found the regulation of DX2 expression to play a critical role in improving PD symptoms, strongly suggesting that DX2 is an effective drug target for PD.

Gene therapy is a therapeutic method of directly injecting a gene to treat a genetic defect, and has been widely developed as a therapeutic option for the treatment of degenerative and intractable diseases [50]. Adeno-associated viruses are currently used for gene therapy to treat PD. Clinical trials using AAV targeting PD-inducing genes, such as AAV2-hGDNF [30, 53] and AAV2-hAADC [3], are currently in progress. Despite concerns about the safety of gene therapy, direct brain administration of AAV has been approved in phase I/II clinical trials of PD [9, 14, 19].

Gene therapy may be a promising strategy for accessing CNS and treating diseases, such as Parkinson’s disease, with minimal invasiveness of intracranial micro-injection. Among the various viral vectors, adeno-associated virus serotype (AAV) is accepted as a suitable gene delivery tool, owing to the following advantages: (1) long-term and stable transgene expression in non-dividing cells following the administration of a single dose and (2) relative safety of use due to the lack of pathogenicity. More than 100 AAV serotypes are known to exist, of which, AAV2 is the most-selected for CNS gene therapy application due to its specific tropism, defined by CNS tissue-specific distribution and low-level systemic distribution [40].

The current study is the first to report that overexpression of DX2 using AAV in the substantia nigra rescues motor activity and neuronal cell death in PD-induced mouse models. Considering the proven safety of AAV in clinical trials, we suggested that AAV-DX2 could be used as a therapeutic drug for patients with PD.

## Conclusion

AIMP2 and DX2 controlled neuronal parthanatos bidirectionally, as an ‘ON/OFF’ switch for PARP-1. DX2-coding self-complementary AAV2 appeared significant therapeutic and neuroprotective effects in chemically-induced Parkinson’s disease animal models.

### Supplementary Information

**Additional file 1.** **Fig. S1. Both AIMP2 and DX2 were ubiquitinated by PARKIN similarly**. Flag-taggedEV, AIMP2 and DX2 were transfected with myc-tagged parkin and HA-tagged ubiquitin vector. Immunoprecipitation was performed using Flag antibody under conditions treated with MG132. Ubi : ubiquitin. **Figure S2. DX2 knock-down with siRNA** SH-SY5Y cells were transfected with scrambled (si-Con) and DX2 small interfering RNA and total RNA in transfected cells were analyzed by quantitative RT-PCR. ***P<0.001, t-test. **Figure S3.Kaplan-Meier graph between WT and DX-TG mice**. There is no different survival rate between WT and DX-TG mice. P : P-value. **Figure S4. Immunofluorescence image of dopaminergic neuron in 6-OHDA induced PD mouse model**. TH positive cells in the substantia nigra of a normal mouse (upper panel). Unilateral loss of TH positive cells in the substantia nigra of the 6-OHDA model (lower panel). **Figure S5. DX2 has no oncogenic characteristic**. A: Representative image of colony forming assay. The colony arised in KRAS overexpressed cells but not DX2-cells. B: Relative colony number graph. ns, not significant.

## Data Availability

All data are available in the main text or the supplementary materials.

## Abbreviations

AIMP2: Aminoacyl tRNA Synthetase Complex Interacting Multifunctional Protein 2
ssAAV: Single strand adeno-associated virus
scAAV: Adeno-associated virus
PARP-1: Poly-(ADP-ribose)-polymerase 1
6-OHDA: 6-Hydroxydopamine hydrobromide
MPTP: 1-Methyl-4-phenyl-1,2,3,6-tetrahydropyridine
L-DOPA: Levodopa
AIF: Apoptosis-inducing factor
PD: Parkinson’s disease
SNpc: Substantia nigra pars compacta
CNS: Central nervous system
EBS: Elevated body swing
WT: Wild type
TG: Transgenic
