# Supplementary material for: Bi-directional regulation of AIMP2 and its splice variant on PARP-1-dependent neuronal cell death; Therapeutic implication for Parkinson's disease
Source: Acta Neuropathol Commun. 2024 Jan 3;12:5. doi: 10.1186/s40478-023-01697-5 (PMC10765824; doi:10.1186/s40478-023-01697-5)
Supplement: Supplementary file 1 — Additional file 1. Fig. S1. Both AIMP2 and DX2 were ubiquitinated by PARKIN similarly. Flag-taggedEV, AIMP2 and DX2 were transfected with myc-tagged parkin and HA-tagged ubiquitin vector. Immunoprecipitation was performed using Flag antibody under conditions treated with MG132. Ubi : ubiquitin. Figure S2. DX2 knock-down with siRNA SH-SY5Y cells were transfected with scrambled (si-Con) and DX2 small interfering RNA and total RNA in transfected cells were analyzed by quantitative RT-PCR. ***P<0.001, t-test. Figure S3.Kaplan-Meier graph between WT and DX-TG mice. There is no different survival rate between WT and DX-TG mice. P : P-value. Figure S4. Immunofluorescence image of dopaminergic neuron in 6-OHDA induced PD mouse model. TH positive cells in the substantia nigra of a normal mouse (upper panel). Unilateral loss of TH positive cells in the substantia nigra of the 6-OHDA model (lower panel). Figure S5. DX2 has no oncogenic characteristic. A: Representative image of colony forming assay. The colony arised in KRAS overexpressed cells but not DX2-cells. B: Relative colony number graph. ns, not significant. [file 40478_2023_1697_MOESM1_ESM.docx]

**Supplementary material**

**Bi-directional regulation of AIMP2 and its splice variant on PARP-1-dependent neuronal cell death; Therapeutic implication for Parkinson's disease**

**Supplementary Method**

**Tumorigenicity assay.**

Anchorage-independent growth was assessed with soft agar colony formation assay using a 6-well plate. The based layer was prepared from a 0.6% soft agar solution containing α-MEM with 10% FBS. Then, 1x10^4^ cells tranfected with DX2, c-Myc, KRAS were suspended in α-MEM containing 10% FBS and 0.35% agar solution and plated onto the base layer. Plates were incubated at 37˚C with 5% CO2 for 21 days, after which colony formation was observed under a microscope.

**Additional file : Supplementary Figures**

**Additional file 1: Fig. S1. Both AIMP2 and DX2 were ubiquitinated by PARKIN similarly.** Flag-tagged EV, AIMP2 and DX2 were transfected with myc-tagged parkin and HA-tagged ubiquitin vector. Immunoprecipitation was performed using a Flag antibody under conditions treated with MG132. Ubi : ubiquitin. **Figure S2. DX2 knock-down with siRNA** SH-SY5Y cells were transfected with scrambled (si-Con) and DX2 small interfering RNA and total RNA in transfected cells were analyzed by quantitative RT-PCR. ****P*<0.001, *t*-test. **Figure S3. Kaplan-Meier graph between WT and DX-TG mice.** There is no different survival rate between WT and DX-TG mice. *P* : *P*-value. **Figure S4. Immunofluorescence image of dopaminergic neuron in 6-OHDA induced PD mouse model.** TH positive cells in the substantia nigra of a normal mouse (upper panel). Unilateral loss of TH positive cells in the substantia nigra of the 6-OHDA model (lower panel). **Figure S5. DX2 has no oncogenic characteristic.** A: Representative image of colony forming assay. colony arised in KRAS overexpressed cells not DX2. B: Relative colony number graph. ns, no significant.

**Supplementary Fig. 1**

**Ubiquitination of AIMP and DX2 by PARKIN**

**
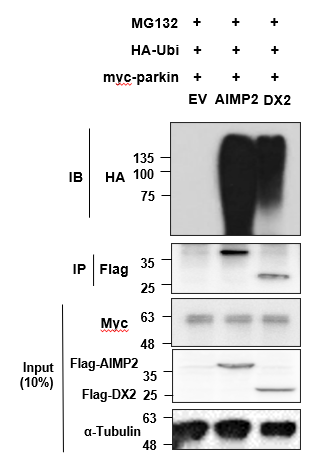
**

**Supplementary Fig. 2**

**DX2 knock-down with siRNA**

*******


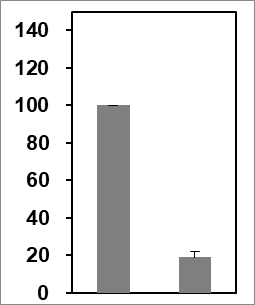


**si-Con**

**si-DX2**

**DX2 expression ratio (%)**

**Supplementary Fig. 3**

**Life span graph between WT and DX2-TG mice**


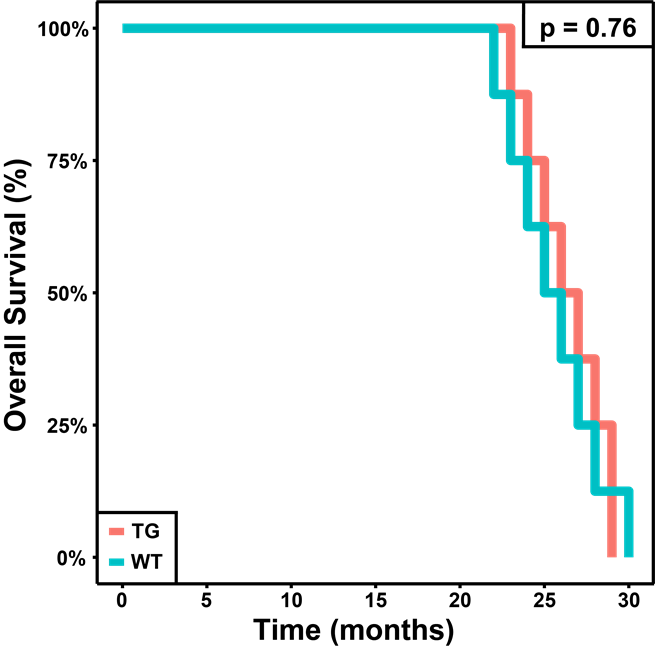


**Supplementary Fig. 4**

**Immunofluorescence image**


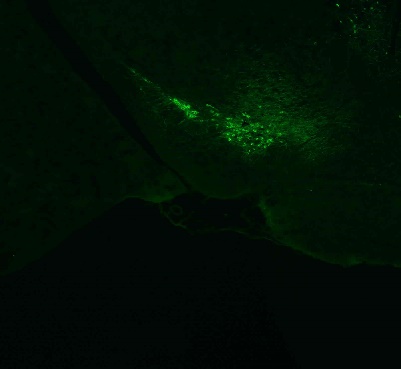

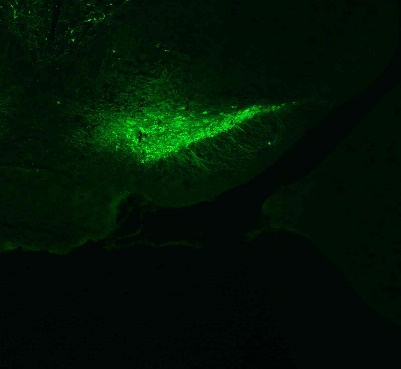

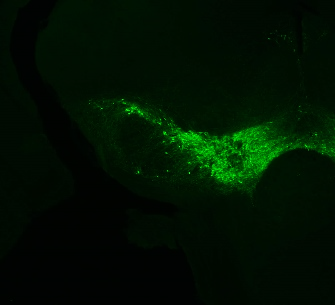

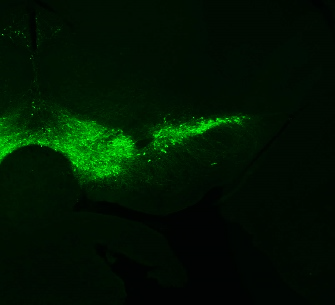


**Substantia nigra**

**Naive**

**6-OHDA**

**MPTP**

**Supplementary Fig. 5**

**B**

**Colony transforming assay**

**
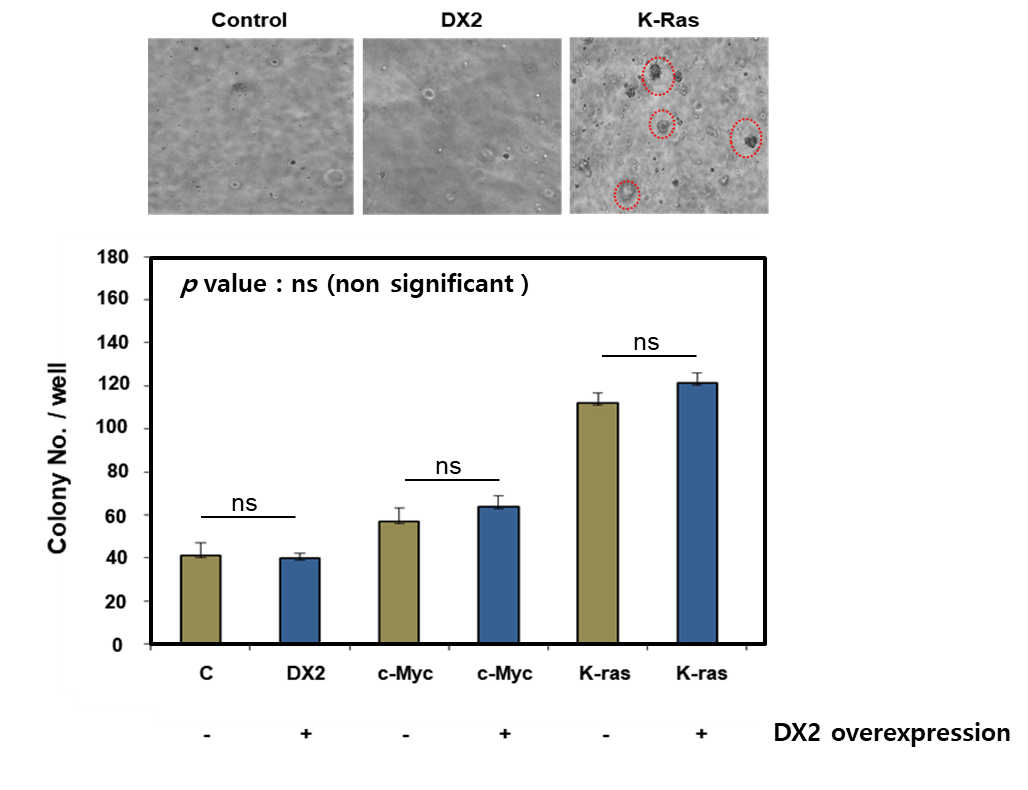
 A**


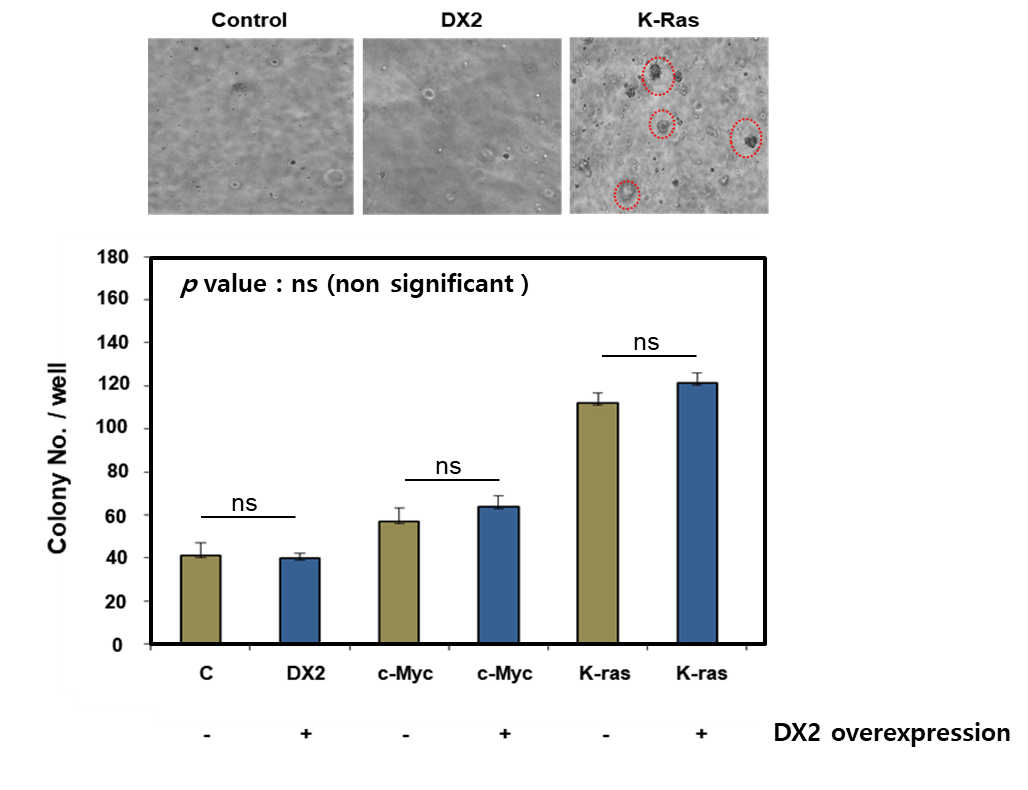
 **B**
